# Characterizing acyl-carnitine biosignatures for schizophrenia: a longitudinal pre- and post-treatment study

**DOI:** 10.1038/s41398-018-0353-x

**Published:** 2019-01-17

**Authors:** Bing Cao, Dongfang Wang, Zihang Pan, Elisa Brietzke, Roger S. McIntyre, Natalie Musial, Rodrigo B. Mansur, Mehala Subramanieapillai, Jing Zeng, Ninghua Huang, Jingyu Wang

**Affiliations:** 1 Department of Laboratorial Science and Technology, School of Public Health, Peking University, Beijing, 100191 P. R. China; 2 Mood Disorders Psychopharmacology Unit, Toronto Western Hospital, University Health Network, Toronto, ON Canada; 3 Department of Psychiatry, Federal University of São Paulo, São Paulo, SP Brazil; 4 Brain and Cognition Discovery Foundation, Toronto, ON Canada; 5 Beijing Key Laboratory of Toxicological Research and Risk Assessment for Food Safety, Beijing, 100191 P.R. China; 6 Peking University Medical and Health Analysis Center, Peking University, Beijing, 100191 P.R. China

## Abstract

Subjects with schizophrenia have high risks of metabolic abnormalities and bioenergetic dysfunction. Acyl-carnitines involved in bioenergetic pathways provide potential biomarker targets for identifying early changes and onset characteristics in subjects with schizophrenia. We measured 29 acyl-carnitine levels within well-characterized plasma samples of adults with schizophrenia and healthy controls using liquid chromatography-mass spectrometry (LC-MS). Subjects with schizophrenia were measured at baseline and after 8 weeks of treatment. A total of 225 subjects with schizophrenia and 175 age- and gender-matched healthy controls were enrolled and 156 subjects completed the 8-week follow-up. With respect to plasma acyl-carnitines, the individuals with schizophrenia at baseline showed significantly higher levels of C4-OH (C3-DC) and C16:1, but lower concentrations of C3, C8, C10, C10:1, C10:2, C12, C14:1-OH, C14:2, and C14:2-OH when compared with healthy controls after controlling for age, sex, body mass index (BMI), smoking, and drinking. For the comparison between pretreatment and posttreatment subjects, all detected acyl-carnitines were significantly different between the two groups. Only the concentration of C3 and C4 were increased after selection by variable importance in projection (VIP) value >1.0 and false discovery rate (FDR) *q* value <0.05. A panel of acyl-carnitines were selected for the ability to differentiate subjects of schizophrenia at baseline from controls, pre- from post-treatment, and posttreatment from controls. Our data implicated acyl-carnitines with abnormalities in cellular bioenergetics of schizophrenia. Therefore, acyl-carnitines can be potential targets for future investigations into their roles in the pathoetiology of schizophrenia.

## Introduction

Schizophrenia is a severe and complex lifelong mental disorder associated with significant morbidity and mortality^1^. The prevalence of schizophrenia is between 0.5 and 1.0% worldwide^1,2^. Schizophrenia is a multifaceted mental illness with both genetic and environmental etiology, characterized by psychopathology, as well as cognitive and neurobiological abnormalities^3^. Despite decades of research, the underling pathogenic pathways underlying schizophrenia has yet to be comprehensively understood. It has been previously established that subjects with schizophrenia have relatively higher rates of metabolic abnormalities and bioenergetic dysfunction (i.e., metabolic syndrome, obesity, and type 2 diabetes mellitus)^4–6^. Further studies suggest that schizophrenia can be characterized by molecular targets, thus presenting opportunities to identify metabolic abnormalities before the initiation of treatment^7,8^.

Carnitines are essential dietary nutrients obtained from meat and dairy products, or are endogenously biosynthesized from L-lysine and L-methionine in the liver and kidneys^9,10^. The optically-active isomer L-carnitine is present in mammalian cells as both free carnitine and acyl-carnitines^11^. Accumulating evidence from animal and human studies indicate that carnitines are quaternary ammonium molecules involved in many metabolic pathways. A recent review paper summarized that acyl-carnitines play an essential role in transporting long-chain fatty acids across the mitochondrial inner membrane during *β*-oxidation, which increases antioxidant activity, stimulates the activity of certain proteins, and enhances cholinergic neurotransmission^11^. Fatty acid metabolism is intricately involved with energy bioenergetics and oxidative pathways^11,12^. Given that carnitines are implicated in fatty acid oxidation, energy production, and cellular homeostasis pathways, the dysfunction of carnitines would be expected to produce a wide variety of disorders affecting the functions of several organs, including, but not limited to, liver, skeletal muscle, heart, and brain^13,14^. A recent animal study with healthy mice reported that chronic acetyl-L-carnitine intake alters brain energy metabolism and increases norepinephrine and serotonin content^15^. In human health and disease research, acyl-carnitine profiles have been widely used to identify metabolic perturbations^11^, including inborn errors of metabolism^16^, thyroid dysfunction^17^, and type 2 diabetes^18^. Carnitine and acyl-carnitine supplementation have also been used in the treatment of various illnesses (e.g., Alzheimer’s disease, peripheral arterial disease, and celiac disease) for decades with the primary function of regulating cellular bioenergetics^19,20^. Furthermore, accumulated evidence indicates that alterations of carnitines may contribute to certain mental illnesses, including depression^21^, autism spectrum disorder^22^, and schizophrenia^23^. In particular, several recent studies have reported that schizophrenia is associated with an imbalance in redox or oxidative stress status. Current replicated evidence suggests that acyl-carnitines are involved in bioenergetics, β-oxidation, and tricarboxylic acid cycle regulation in the mitochondrion. These mechanism could play important roles in the pathogenesis of schizophrenia^24,25^. In addition, acetyl-carnitine and levocarnitine^26,27^ supplementation therapies have been used to target carnitine deficiency observed in individuals with schizophrenia. Available evidence suggest that acyl-carnitines impact insulin sensitivity, lipid metabolism, inflammatory response, as well as bioenergetic functions^23,28^. Based on these foregoing evidence, we believe acyl-carnitines play an essential role in the pathoetiology of schizophrenia. Thus, a comprehensive analysis of acyl-carnitine profiles in a treatment population of individuals with schizophrenia was conducted.

The hypothesis of the current study is that bioenergetic abnormalities defined by acyl-carnitine imbalance, exist in subjects with schizophrenia. By utilizing a targeted metabolic profiling approach, our current study was able to accurately quantify acyl-carnitine profiles in plasma samples among individuals with schizophrenia at baseline and after 8 weeks of antipsychotic therapy. The same metabolic profiling approach was applied to the healthy control subjects. Herein, our study aimed to find potential abnormalities of metabolic pathways involving acyl-carnitines to further explore the connection between schizophrenia and metabolism.

## Methods

### Study population

Two hundred twenty-five individuals with a primary diagnosis of schizophrenia according to the Diagnostic and Statistical Manual of Mental Disorders, fourth edition (DSM-IV) were enrolled. All subjects were inpatients from the Mental Health Center of Weifang (Shandong Province, China). All subjects received antipsychotic treatment and were invited to an 8-week follow-up survey. Subjects received antipsychotic therapies according to their clinical performance. Among the 225 subjects enrolled, 69 withdrew or were lost to follow-up. During the same time period, 175 healthy individuals from the same city district as the subjects with schizophrenia, that are age (± 5 years), sex, and ethnicity matched, without a history of DSM-defined psychiatric disorders, were enrolled as a control comparison group. All subjects met the following criteria: (1) age from 18 to 60 years old; (2) absence of diabetes mellitus, hyperlipidemia, cardiovascular disease, or any other severe or unstable medical illness; (3) absence of comorbidity with other psychiatric disorders, including alcohol and substance use disorders. All subjects were either first psychotic episode and drug-naive or had recurrent schizophrenia and had not taken any antipsychotic drugs for a minimum of 4 weeks before hospitalization. Clinical assessments took place between November 2015 and September 2016.

Data on basic demographic characteristics was collected by a standardized questionnaire. The Case Report Form (CRF) was used to collect clinical data, clinical history, family history, and medication records. The Positive and Negative Syndrome Scale (PANSS) scores were assessed during admission/before treatment (week 0) and after treatment (week 8). Treatment medication details are shown in Table S1. During the trial, all subjects received a standard diet provided by the hospital and abstained from alcohol and smoking. For the control group, basic clinical information and blood samples were collected for one time only (i.e., at week 0).

### Ethical approval

The research ethics committee of the Peking University Health Science Center (Beijing, China) reviewed and approved the study protocols (IRB00001052-14071). All subjects provided written informed consent prior to their inclusion. All procedures were performed in accordance with the Helsinki Declaration standards as revised in 1989.

### Plasma samples preparation

All fasting blood samples (~5 mL) were collected from subjects at baseline (week 0) before initiation of antipsychotic treatment and after 8 weeks of antipsychotic treatment following overnight fasting (12 h fasting, collected between 7 and 9 am). Samples were maintained at 4 ℃ for 20–30 min and the resulting plasma aliquot was transferred into Eppendorf tubes, which were subsequently stored at −80 ℃. The plasma samples of the control group were collected with the same protocols as the schizophrenia group.

### Chemicals and reagents

Analytical reference standards of 29 acyl-carnitines (Sigma-Aldrich, Co., St. Louis, MO, USA) were employed for quality control of compounds in samples. We defined acyl-carnitines and major acyl-carnitine fractions as free-, short-, medium-, or long-chain acyl-carnitines according to the length of carbon chains in the molecular structure (i.e., free carnitine: C0, short-chain: C2-C5, medium-chain: C6-C12, long-chain: C14-C18)^29,30^. Deuterium-labeled carnitine and acyl-carnitines (NSK-B Set, Cambridge Isotope Laboratories, Inc., Tewksbury, MA, USA) were used as internal standards, including ^2^H_9_-carnitine (free carnitine, CN), ^2^H_3_-acetylcarnitine (C2), ^2^H_3_-propionylcarnitine (C3), ^2^H_3_-butyrylcarnitine (C4), ^2^H_9_-isovalerylcarnitine (C5), ^2^H_3_-octanoylcarnitine (C8), ^2^H_9_-myristoylcarnitine (C14), and ^2^H_3_-palmitoylcarnitine (C16). HPLC-grade ammonium acetate was purchased from Sigma-Aldrich (St. Louis, MO, USA); HPLC-grade formic acid and methanol were supplied by Thermo Fisher Scientific (Waltham, MA, USA); pure water was purchased from Wahaha Co., Ltd. (Hangzhou, China).

### Carnitine and acyl-carnitines extraction from plasma samples

The deuterium-labeled carnitine and acyl-carnitines were initially redissolved in 1 mL methanol as stock solution for internal standards quantification. Mixtures of 45 μL thawed plasma and 135 μL of methanol solution (1:3, v/v) spiked with 5.4 μL of isotopically labeled internal standards (v/v = 24/1) were vibrated and centrifuged (4 ℃, 12,000 rpm for 10 min) and 100 µL of supernatant was collected into a 200 µL vial insert for analysis. Equal volumes of the supernatant from all samples were transferred each time to a limited volume vial. Pooled quality control samples (QCs) consisted of small aliquots of each biological sample being pooled and mixed together to monitor the stability and repeatability of the results.

### HILIC mode and UHPLC-MS analysis

Plasma acyl-carnitine profiles were measured in a randomized order. The UPLC separation was performed using the Thermo Scientific™ Dionex™ UltiMate™ 3000 Rapid Separation LC (RSLC) system with a BEH Amide column (2.1 × 100 mm, 1.7 μm; Waters Corp). Samples were analyzed using a positive ionization mode (ESI+) with the following settings: the column flow rate was 300 µL/min, the column temperature was set at 50 °C, the auto-sampler temperature was kept at 4 °C, and the injection volume was 1 µL. The working solvent A was methanol and solvent B was ultrapure water; both A and B contained 0.1% formic acid and 10 mmol/L ammonium acetate. The separation gradient used was: 0–1 min (95.0% A), 1–10 min: (95.0–50.0% A), 10–12 min: (50.0% A), 12–12.1 min (50.0–95.0% A), and 12.1–16 min: (95.0% A).

After the chromatographic separation, MS ionization and data acquisition were performed using a Thermo Scientific™ Q Exactive™ hybrid quadrupole Orbitrap mass spectrometer equipped with a HESI-II probe. The HESI-II spray voltages were 3.7 kV. The heated capillary temperature was 320 °C, the sheath gas pressure was 30 psi, the auxiliary gas setting was 10 psi, and the heated vaporizer temperature was 300 °C. Both the sheath gas and the auxiliary gas were nitrogen. The collision gas was also nitrogen at a pressure of 1.5 mTorr. The parameters for the full mass scan were as follows: a resolution of 70,000, an auto gain control target under 1 × 10^6^, a maximum isolation time of 50 ms, and an *m*/*z* range of 70–1050. The calibration was customized for the Q Exactive to keep the mass tolerance of 5 ppm. The parameters for the QC-MS2 scan were as follows: a resolution of 17,500, an auto gain control target under 1 × 10^5^, a maximum isolation time of 50 ms, a loop count of top 10 peaks, an isolation window of *m*/*z* 2, a normalized collision energy of 30 V, and an intensity threshold under 1 × 10^5^. The final LC-MS method employed for the quantification of carnitines and internal standards was composed of 29 carnitines and 8 internal standards ESI+ timed functions properly segmented over the 10 min chromatographic run. The HILIC UPLC-MS system control and data acquisition were performed with the Thermo Fisher Corporation Xcalibur 2.2 SP1.48 software (Waltham, MA). Skyline (64-bit, 3.5.0.9319, MacCoss Lab, UW) was used to visualize, process, and interpret the MS raw data, allowing for the discovery and identification of carnitines among sample groups.

### Statistical analysis

All plasma acyl-carnitine levels were quality reassured by analytical reference standards, quantified and normalized by its corresponding internal standard level (Table S2), and log_10_-transformed to approximate normality. Descriptive statistics were performed for the basic characteristics and acyl-carnitine analysis consisted of categorical variables summarized as frequencies and proportions, while continuous variables were summarized as the mean and standard deviation (SD) or median and interquartile range (IQR). For categorical variables, statistical significance between various groups was tested using the *χ*^2^ test. For continuous variables, according to the features of data, the independent Student’s *t*-test or Mann–Whitney U test was used for comparisons between the two independent groups (i.e., control and patient groups, or first and recurrent subjects). The paired Student’s *t*-test or Wilcoxon signed-rank *t*-test was employed for comparisons between the two related groups (i.e., pre- and post-treatment groups). A two-sided *P* < 0.05 was considered as statistically significant. The Benjamini–Hochberg false discovery rate (FDR) control was implemented to correct for multiple comparisons of 29 acyl-carnitines. In this study, the FDR *q*-value threshold for significant markers was set at 0.05.

Orthogonal partial least-squares discriminant analysis (OPLS-DA) was performed using log_10_-transformed and auto-scaled data to construct classification models. An internal 7-fold cross-validation was carried out to estimate the performance of the OPLS-DA model. Model validation was performed using 300-iteration permutation tests. R^2^ represents the explanation capacity of the model, while Q^2^ stands for the predictive capacity of the model. OPLS-DA model was performed by the SIMCA-P software 14.1 (Umetrics, Umeå, Sweden).

Based on the *q* value < 0.05 from univariate analysis and the highest variable importance in projection (VIP) value >1.0 from the OPLS-DA model, several metabolites were selected as a panel of markers to differentiate subjects of pretreatment, posttreatment patients, and healthy controls. Partial correlation analysis on ranks (Spearman Correlation) was used to calculate correlation coefficients among acyl-carnitines and between clinical characteristics with acyl-carnitines, which was conducted by Metaboanalyst 3.0 software. Generalized linear models (GLM) were applied to comparisons of carnitines’ levels between two groups with adjustment for confounding factors. According to the previous metabolic profiling research^31,32^, age, sex, BMI, smoking, and drinking were defined as potential confounders in our data analysis. Due to the potential linearity and collinearity, integrating individual carnitine was done one at a time into the GLM. Area under the receiver-operating characteristic (ROC) curve (AUROC) was then calculated to evaluate its classification performance using SPSS 22.0 (SPSS Inc., Chicago, IL, USA). Backward stepwise logistic regression models based on Akaike’s information criterion (AIC)^33^ were also performed by SPSS 22.0 to optimize the metabolite biomarker combination for discrimination between pretreatment and posttreatment schizophrenia subjects, as well as healthy controls.

## Results

### Demographic and clinical characteristics of the sample

In total, 225 individuals with schizophrenia and 175 healthy controls were included in the analysis. Demographic data and clinical characteristics were compared between individuals with schizophrenia at baseline and healthy controls (Table 1). Individuals at baseline with schizophrenia were younger than controls (*P* = 0.04). The mean age of subjects and controls were 37.31 years (SD = 10.85 years) and 39.44 years (SD = 9.36 years), respectively. In terms of gender distribution, 60.0% of the subjects and 69.1% of the healthy controls were female. Of the individuals with schizophrenia, the mean age of onset was 22.74 (SD = 9.70) years, while the mean duration of illness was 4.48 (SD = 2.67) years. Of the 225 subjects with schizophrenia, 40 were first-episode and treatment-naive, while the remaining 185 schizophrenia subjects were recurrent (had psychotic episodes in the past) and had not taken any antipsychotic drugs for at least 1 month prior to hospitalization. No significant changes in BMI, fasting blood glucose (FBG), total cholesterol (TC), triglycerides (TG) and very low-density lipoprotein (VLDL) were observed between the two groups (all *P* > 0.05). The comparison of all basic characteristics between first episode and recurrent episode patients before and after 8-week follow-up are shown in Table S4.

**Table 1:** Baseline characteristics of subjects with schizophrenia at baseline and healthy control

Variables	Schizophrenia	Control	*P* value
	*n* *=* 225	*n* = 175	
Age (years); mean (SD)	37.31 (10.85)	39.44 (9.36)	0.040^a^
Gender (female); *n* (%)	135 (60.0)	121 (69.1)	0.061^b^
Smoker; *n* (%)	25 (11.3)	21 (12.0)	0.936^b^
Drinker; *n* (%)	10 (5.0)	30 (17.4)	<0.001^b^
Age of onset (years); mean (SD)	27.74 (9.70)	–	–
Duration of illness (years); mean (SD)	4.48 (2.67)	–	–
First-episode subjects; *n* (%)	40 (17.78)	–	–
BMI (kg/m^2^); mean (SD)	24.11 (3.85)	23.91 (3.14)	0.596^a^
FBG (mmol/L); mean (SD)	5.73 (1.78)	5.70 (1.02)	0.869^a^
TG (mmol/L); mean (SD)	1.25 (0.9)	1.17 (0.8)	0.354^a^
TC (mmol/L); mean (SD)	4.66 (1.12)	4.76 (0.97)	0.368^a^
VLDL (mmol/L); mean (SD)	0.58 (0.41)	0.54 (0.35)	0.308^a^

*BMI* body mass index, *FBG* fasting blood glucose, *TG* triglyceride, *TC* total cholesterol, *VLDL* very low density lipoprotein, *SD* standard deviation

^a^*P* values were calculated by two-tailed *t*-tests

^b^*P* values were calculated by chi-square tests

Before the 8-week follow-up point, 69 of the 225 subjects withdrew from the study (either refused to follow-up or lost contact) at various points. Comparison of the clinical characteristics of the remaining 156 pretreatment and posttreatment subjects was completed after 8 weeks (Table 2). The scores of all PANSS domains were significantly decreased after treatment (*P* < 0.05) indicating a reduction of schizophrenia severity. No significant change from baseline in TC and low-density lipoprotein (LDL) were observed after the follow-up at 8 weeks (all *P* > 0.05), while the BMI, waist circumference, TG, and VLDL were increased after treatment. FBG and high-density lipoprotein (HDL) were decreased after treatment.

**Table 2:** Demographic and clinical characteristics of pretreatment and posttreatment subjects

Variables	Pretreatment	Posttreatment	*P* value^a^
	*n* = 156	*n* = 156	
PANSS scores; mean (SD)			
PANSS total	88.82 (18.23)	50.67 (12.7)	**<** **0.001**
PANSS positive	21.78 (8.09)	9.96 (4.00)	**<** **0.001**
PANSS negative	21.15 (7.99)	13.65 (5.25)	**<** **0.001**
General psychopathology	42.84 (12.49)	25.67 (7.94)	**<** **0.001**
BMI (kg/m^2^); mean (SD)	23.89 (3.96)	24.70 (3.70)	**<** **0.001**
Waist (cm); mean (SD)	88.71 (12.19)	90.63 (11.20)	**<** **0.001**
FBG (mmol/L); mean (SD)	5.62 (1.80)	5.21 (1.00)	**0.005**
TG (mmol/L); mean (SD)	1.24 (0.82)	1.96 (1.15)	**<** **0.001**
TC(mmol/L); mean (SD)	4.61 (1.08)	4.63 (0.92)	0.821
HDL (mmol/L); mean (SD)	1.42 (0.30)	1.35 (0.32)	**0.012**
LDL (mmol/L); mean (SD)	2.25 (0.45)	2.29 (0.46)	0.295
VLDL (mmol/L); mean (SD)	0.57 (0.38)	0.91 (0.52)	**<** **0.001**

*BMI* body mass index, FBG fasting blood glucose, *TG* triglyceride, *TC* total cholesterol, *HDL* high-density lipoprotein, *LDL* low density lipoprotein, *VLDL* very low density lipoprotein, *SD* standard deviation

^a^*P* values were calculated by two-tailed paired-samples

### Plasma acyl-carnitine profiles of subjects

**Fig. 1:**
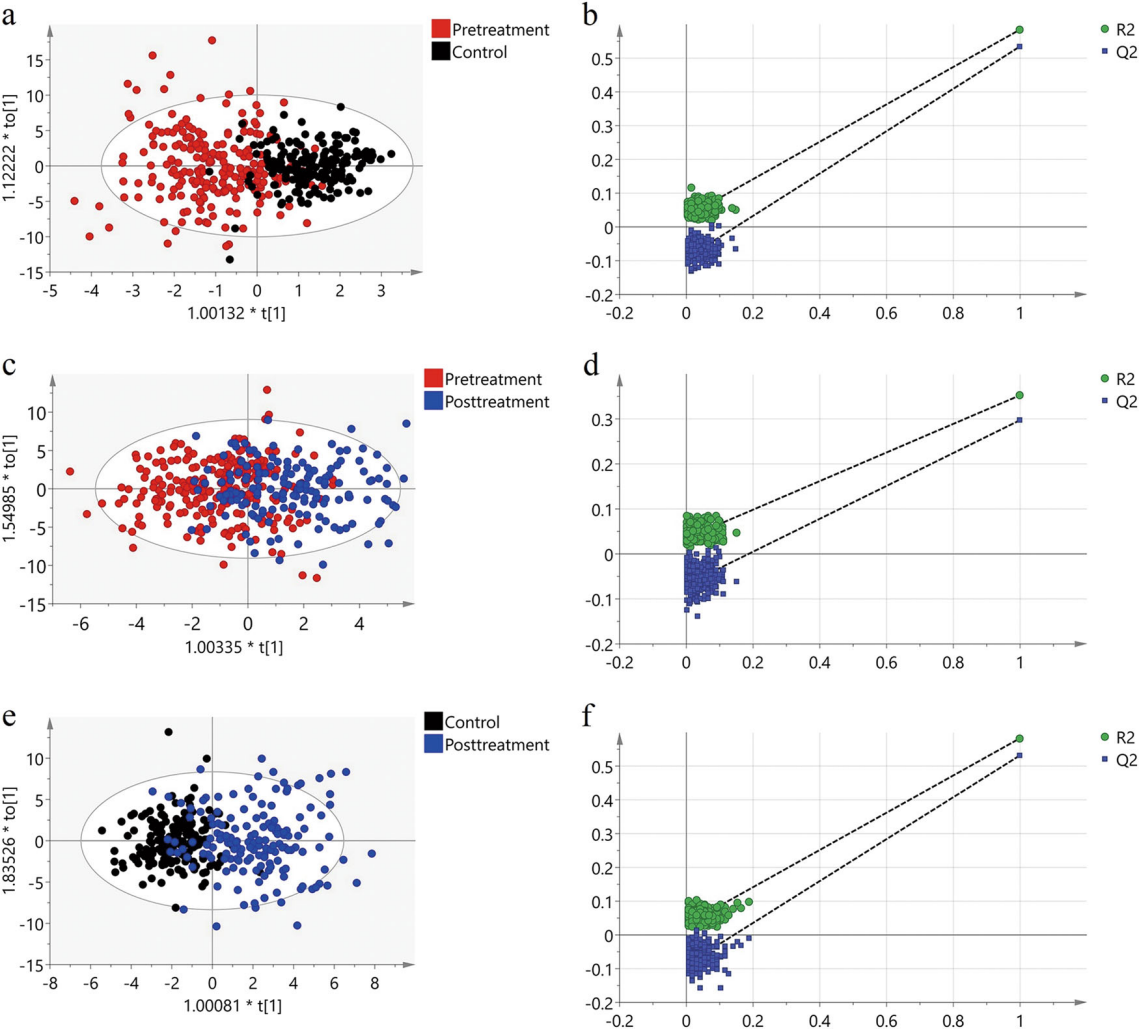
Box plots of differential carnitines for the comparison between subjects at baseline and controls or upon treatment. The *X*-axis is the acyl-carnitines, and the *Y*-axis is the concentration of acyl-carnitines (µmol/L). *Statistically significant with *P* < 0.05 in the comparison between subjects at baseline and controls. ^#^Statistically significant with *P* < 0.05 in the comparison between posttreatment subjects and controls

**Fig. 2:**
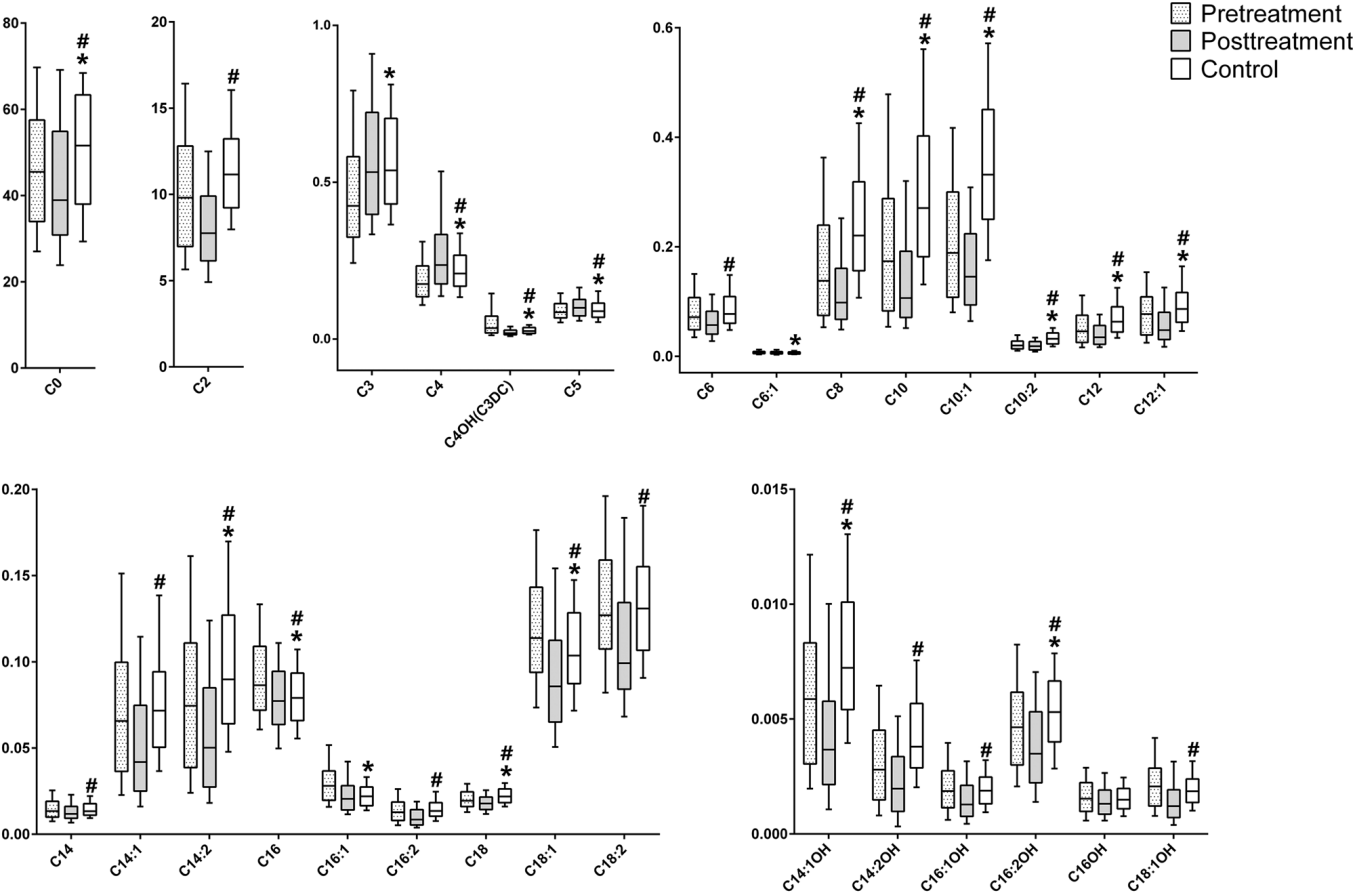
Orthogonal projections to latent structures discriminant analysis (OPLS-DA) for healthy controls, pretreatment subjects, and posttreatment subjects. **a** Score plot of the OPLS-DA model for the discrimination of healthy controls and subjects with schizophrenia at baseline, R^2^X (cum) = 0.832, R^2^Y (cum) = 0.585, Q^2^ (cum) = 0.535. **b** Statistical validation of the OPLS-DA model in (**a**) by permutation testing. **c** Score plot of the OPLS-DA model for the differentiation of pretreatment and posttreatment subjects, R^2^X (cum) = 0.775, R^2^Y (cum) = 0.352, Q^2^ (cum) = 0.297. **d** Statistical validation of the OPLS-DA model in (**c**) by permutation testing. **e** Score plot of the OPLS-DA model for the differentiation of healthy controls and posttreatment subjects, R^2^X (cum) = 0.772, R^2^Y (cum) = 0.583, Q^2^ (cum) = 0.532. **f** Statistical validation of the OPLS-DA model in (**e**) by permutation testing

Plasma acyl-carnitine levels of each group are illustrated in Fig. 1 and Table S3. All OPLS-DA models between the two groups indicated adequate classification (Fig. 2a–e). There were 11 out of 29 acyl-carnitines altered in subjects with schizophrenia at baseline compared to controls (FDR *q* < 0.05 and OPLS-DA VIP > 1.0, shown in Table 3). The subjects with schizophrenia at baseline showed significantly higher levels of C4-OH(C3-DC) and C16:1, but lower concentrations of C3, C8, C10, C10:1, C10:2, C12, C14:1-OH, C14:2, and C14:2-OH. All the differences still have statistical significance after controlling for age, sex, BMI, smoking, and drinking. After administration of the 8-week clinical therapy, all detected acyl-carnitines had significant differences between the pre- and post-treatment groups with FDR *q* *<* 0.05. Thirteen acyl-carnitines have VIP > 1.0 with the OPLS-DA selection. Only the concentration of C3 and C4 were increased while all the remaining 11 acyl-carnitines were decreased. We also conducted the comparison between posttreatment patients and controls to explore the difference. Our results indicated that all the fourteen selected acyl-carnitines with FDR *q* < 0.05 and OPLS-DA VIP > 1.0 (i.e., C2, C8, C10, C10:1, C10:2, C12, C12:1, C14:1, C14:1-OH, C14:2, C14:2-OH, C16:2, C16:2-OH, and C18) were lower in posttreatment patients after controlling age, sex, BMI, smoking, and drinking (Tables 3 and 4). To validate the reliability of the prediction model, permutation test (*n* = 300) was calculated (Fig. 2b–f). The true-class Q^2^ and R^2^ values were significantly higher than the corresponding permutated values in all three figures, indicating that the model is statistically sound; that its high predictability is not due to over-fitting of the data.

**Table 3:** Comparison of acyl-carnitines between subjects with schizophrenia at baseline vs. healthy controls, pretreatment vs. posttreatment patients, posttreatment patients vs. healthy controls

Category	Patients at baseline vs.			Pretreatment vs.			Posttreatment patients vs.		
	Healthy controls			Posttreatment patients			Healthy controls		
	*q* ^a^	FC^b^	VIP	*q* ^c^	FC^d^	VIP	*q* ^e^	FC^f^	VIP
C0	7.87E−04	0.86	0.96	0.03	0.84	0.78	1.22E−07	0.72	0.93
C2	0.47	0.93	0.69	3.44E−08	0.74	1.18	6.06E−21	0.68	1.15
C3	1.56E−10	0.76	1.29	1.74E−09	1.31	1.15	0.92	1.02	0.67
C4	1.50E−04	0.81	0.99	4.54E−11	1.47	1.28	1.81E−06	1.19	0.81
C4-OH(C3-DC) (10^−2^)	1.56E−06	1.39	1.04	2.53E−13	0.51	1.27	1.83E−07	0.71	0.81
C5 (10^−2^)	0.08	0.95	0.73	1.93E−03	1.2	0.83	0.21	1.11	0.65
C6 (10^−2^)	0.01	0.92	0.86	7.05E−04	0.77	0.97	5.95E−12	0.71	0.98
C6:1 (10^−2^)	0.01	1.11	0.72	1.85E−03	0.87	0.72	0.39	0.94	0.6
C8 (10^−2^)	3.39E−11	0.59	1.34	3.79E−03	0.66	0.93	2.44E−24	0.39	1.25
C10 (10^−2^)	3.28E−11	0.62	1.33	7.05E−04	0.54	0.97	4.87E−26	0.35	1.3
C10:1 (10^−2^)	1.60E−17	0.56	1.55	2.43E−04	0.66	1.02	3.75E−33	0.38	1.37
C10:2 (10^−2^)	2.78E−19	0.59	1.57	0.04	0.87	0.79	1.86E−24	0.52	1.22
C12 (10^−2^)	3.48E−08	0.69	1.19	0.01	0.71	0.9	2.52E−18	0.52	1.15
C12:1 (10^−2^)	1.25E−03	0.91	0.96	1.63E−04	0.56	1.03	3.10E−15	0.53	1.12
C14 (10^−2^)	0.58	1.01	0.74	7.84E−03	0.86	0.85	2.20E−04	0.87	0.84
C14:1 (10^−2^)	0.07	0.93	0.82	1.59E−05	0.59	1.09	4.00E−13	0.55	1.07
C14:1-OH (10^−2^)	6.33E−07	0.8	1.09	4.30E−05	0.61	1.04	2.75E−20	0.49	1.19
C14:2 (10^−2^)	7.71E−05	0.84	1.02	1.06E−04	0.59	1.08	3.20E−18	0.5	1.16
C14:2-OH (10^−2^)	3.63E−09	0.72	1.2	7.05E−04	0.68	0.96	1.02E−19	0.49	1.17
C16 (10^−2^)	1.04E−03	1.09	0.89	8.04E−07	0.82	0.87	0.15	0.96	0.68
C16:1 (10^−2^)	8.73E−06	1.28	1.03	1.14E−08	0.68	1.1	0.03	0.91	0.78
C16:1-OH (10^−2^)	0.97	0.98	0.71	9.36E−05	0.68	1	1.22E−07	0.68	0.91
C16:2 (10^−2^)	0.21	1.04	0.79	4.34E−07	0.59	1.17	3.10E−15	0.6	1.11
C16:2-OH (10^−2^)	0.01	0.89	0.8	2.20E−04	0.71	0.97	2.03E−12	0.64	1.03
C16-OH (10^−2^)	0.53	1.00	0.68	0.02	0.87	0.78	7.12E−05	0.87	0.75
C18 (10^−2^)	1.25E−03	0.89	0.88	9.99E−08	0.86	0.83	3.10E−15	0.76	1.08
C18:1 (10^−2^)	7.87E−03	1.12	0.86	2.53E−13	0.67	1.16	1.22E−07	0.79	0.89
C18:1-OH (10^−2^)	0.94	1.11	0.71	1.37E−06	0.57	1.11	5.91E−10	0.63	0.94
C18:2 (10^−2^)	0.94	0.98	0.55	2.19E−07	0.74	0.84	1.47E−05	0.76	0.76

^a,e^*q* values were FDR corrections for *p*-values which were calculated from two-tailed independent *t*-test of log_10_-transformed data

^b^Fold changes were calculated as the ratios of median metabolite levels (patients at baseline/healthy controls)

^c^*q* values were FDR corrections for *p* values which were calculated from two-tailed paired *t*-test of log_10_-transformed data

^d^Fold changes were calculated as the ratios of median metabolite levels (posttreatment patients/pretreatment patients)

^f^Fold changes were calculated as the ratios of median metabolite levels (posttreatment patients/healthy controls)

**Table 4:** GLM and ROC for comparison of selected acyl-carnitines levels between subjects with schizophrenia at baseline vs. healthy controls, pretreatment vs. posttreatment patients, posttreatment patients vs. healthy controls

Variables	Acyl-carnitines (µmol/L);	median (IQR)	GLM			ROC			
			*β*	95% CI	Z*	AUROC	95% CI	Sensitivity	Specificity
*Patients at baseline* (*n = 225*) *vs. Healthy controls* (*n = 175*)									
C3	0.42(0.31–0.59)	0.55(0.45–0.72)	−0.857^a^	−1.113,−0.601	−6.56	0.692	0.640,0.743	0.714	0.622
C4-OH(C3-DC) (10^−2^)	3.57(1.9–7.85)	2.56(1.79–3.68)	0.317^a^	0.186,0.448	4.75	0.624	0.569,0.679	0.467	0.834
C8 (10^−2^)	13.68(7.25–24.2)	23.1(15.98–34.69)	−0.43^a^	−0.569,−0.29	−6.04	0.702	0.651,0.752	0.789	0.569
C10 (10^−2^)	17.2(7.86–29.64)	27.84(18.44–41.46)	−0.421^a^	−0.553,−0.288	−6.22	0.696	0.645,0.747	0.829	0.507
C10:1 (10^−2^)	18.9(10.54–30.6)	33.97(25.19–46.99)	−0.633^a^	−0.785,−0.482	−8.21	0.755	0.708,0.801	0.777	0.640
C10:2 (10^−2^)	1.95(1.34–2.77)	3.3(2.31–4.46)	−0.852^a^	−1.036,−0.668	−9.09	0.763	0.717,0.808	0.834	0.560
C12 (10^−2^)	4.49(2.42–7.55)	6.48(4.57–9.3)	−0.42^a^	−0.573,−0.266	−5.35	0.657	0.605,0.71	0.714	0.547
C14:1-OH (10^−2^)	0.59(0.3–0.86)	0.74(0.54–1.03)	−0.451^a^	−0.61,−0.292	−5.57	0.633	0.579,0.686	0.617	0.564
C14:2 (10^−2^)	7.58(3.75-11.53)	8.98(6.42–12.8)	−0.341^a^	−0.505,−0.177	−4.08	0.607	0.552,0.662	0.789	0.422
C14:2-OH (10^−2^)	0.28(0.15–0.45)	0.39(0.29–0.58)	−0.453^a^	−0.587,−0.318	−6.61	0.664	0.611,0.716	0.743	0.533
C16:1 (10^−2^)	2.81(1.96–3.82)	2.19(1.65–2.77)	0.509^a^	0.277,0.741	4.31	0.658	0.605,0.711	0.538	0.743
*Pretreatment* (*n* *=* *156*) *vs. Posttreatment patients* (*n* *=* 156)									
C2	10.29(6.92–15.18)	7.62(5.98–9.78)	0.145^b^	0.098,0.191	6.13	0.677	0.618,0.736	0.628	0.628
C3	0.42(0.31–0.58)	0.55(0.40–0.77)	−0.136^b^	−0.175,−0.096	−6.78	0.669	0.610, 0.729	0.654	0.622
C4	0.17(0.13–0.23)	0.25(0.18–0.40)	−0.213^b^	−0.269,−0.157	−7.5	0.708	0.651,0.766	0.603	0.718
C4-OH(C3-DC) (10^−2^)	3.62(1.95–7.81)	1.83(1.34–2.79)	0.349^b^	0.268,0.429	8.47	0.734	0.678,0.79	0.622	0.769
C10:1 (10^−2^)	19.78(10.51–31.22)	13.07(8.35–21.66)	0.148^b^	0.073,0.223	3.89	0.632	0.57,0.693	0.590	0.603
C12:1 (10^−2^)	8.38(3.97–11.83)	4.67(2.75–8.13)	0.162^b^	0.083,0.241	4.02	0.64	0.578,0.701	0.571	0.699
C14:1 (10^−2^)	6.85(3.63–10.23)	4.01(2.3–7.23)	0.188^b^	0.109,0.267	4.67	0.655	0.594,0.715	0.609	0.660
C14:1-OH (10^−2^)	0.59(0.28–0.83)	0.36(0.2–0.57)	0.191^b^	0.106,0.276	4.41	0.646	0.585,0.708	0.603	0.705
C14:2 (10^−2^)	7.62(3.68–11.4)	4.5(2.48–8.41)	0.173^b^	0.091,0.254	4.15	0.641	0.58,0.703	0.609	0.635
C16:1 (10^−2^)	2.92(1.97–3.98)	1.99(1.34–2.77)	0.159^b^	0.11,0.208	6.38	0.696	0.637,0.754	0.705	0.603
C16:1-OH (10^−2^)	0.19(0.11–0.3)	0.13(0.07–0.21)	0.192^b^	0.102,0.281	4.2	0.64	0.579,0.701	0.705	0.526
C16:2 (10^−2^)	1.37(0.83–1.9)	0.81(0.48–1.4)	0.202^b^	0.13,0.274	5.53	0.677	0.618,0.736	0.609	0.654
C18:1 (10^−2^)	12.23(9.84–15.07)	8.2(5.62–11.2)	0.159^b^	0.123,0.196	8.58	0.734	0.678,0.79	0.788	0.615
C18:1-OH (10^−2^)	0.21(0.12–0.3)	0.12(0.06–0.19)	0.249^b^	0.156,0.342	5.24	0.688	0.629,0.746	0.712	0.577
*Posttreatment patients* (*n* = *156*) *vs*. *Healthy controls* (*n* **=** *175*)									
C2	7.62(5.98–9.78)	11.16(9.26–13.24)	−1.404^c^	−1.696,−1.112	−9.44	0.788	0.739,0.838	0.857	0.628
C8 (10^−2^)	9.11(6.19–16.04)	23.1(15.98–34.69)	−0.731^c^	−0.864,−0.598	−10.76	0.822	0.776,0.868	0.777	0.737
C10 (10^−2^)	9.61(6.58–18.74)	27.84(18.44–41.46)	−0.702^c^	−0.823,−0.582	−11.44	0.827	0.781,0.872	0.834	0.705
C10:1 (10^−2^)	13.07(8.35–21.66)	33.97(25.19–46.99)	−0.866^c^	−0.993,−0.74	−13.4	0.867	0.828,0.906	0.806	0.827
C10:2 (10^−2^)	1.71(1.06–2.66)	3.3(2.31–4.46)	−0.949^c^	−1.114,−0.783	−11.22	0.808	0.762,0.854	0.789	0.699
C12 (10^−2^)	3.35(2.05–5.62)	6.48(4.57–9.3)	−0.718^c^	−0.871,−0.565	−9.2	0.776	0.725,0.826	0.720	0.718
C12:1 (10^−2^)	4.67(2.75–8.13)	8.74(6.23–12)	−0.634^c^	−0.792,−0.476	−7.88	0.74	0.685,0.795	0.817	0.596
C14:1 (10^−2^)	4.01(2.3–7.23)	7.23(5.04–9.51)	−0.578^c^	−0.734,−0.423	−7.29	0.718	0.662,0.775	0.783	0.590
C14:1-OH (10^−2^)	0.36(0.2–0.57)	0.74(0.54–1.03)	−0.659^c^	−0.789,−0.528	−9.88	0.787	0.737,0.837	0.766	0.737
C14:2 (10^−2^)	4.5(2.48–8.41)	8.98(6.42–12.8)	−0.659^c^	−0.804,−0.514	−8.93	0.761	0.708,0.814	0.789	0.654
C14:2-OH (10^−2^)	0.19(0.09–0.34)	0.39(0.29–0.58)	−0.547^c^	−0.658,−0.437	−9.71	0.779	0.728,0.831	0.760	0.699
C16:2 (10^−2^)	0.81(0.48–1.4)	1.36(1.04–1.84)	−0.668^c^	−0.835,−0.5	−7.81	0.739	0.684,0.794	0.714	0.686
C16:2-OH (10^−2^)	0.34(0.22–0.53)	0.53(0.4–0.68)	−0.683^c^	−0.861,−0.505	−7.52	0.718	0.662,0.775	0.720	0.660
C18 (10^−2^)	1.69(1.28–2.11)	2.22(1.84–2.66)	−1.458^c^	−1.785,−1.132	−8.76	0.738	0.685,0.792	0.686	0.641

*β* regression coefficient, *CI* confidence interval

*All *P* values have FDR-corrected statistical significance

^a,c^*β* was calculated from GLM with adjustment for age, gender, BMI, current smoking and current drinking.

^b^*β* was calculated from GLM without adjustment

Multivariate analysis for recurrent and first episode subjects showed only C3, C4, and C16 were higher in the recurrent schizophrenia group at baseline; C3, C4, and C5 were higher in the recurrent group after 8-week follow-up, with *P* values < 0.05 after adjusting for age, sex, BMI, smoking, and drinking. After FDR correction, all the acyl-carnitines expressed *q*-values > 0.05 at baseline and 8-week follow-up between the first-episode and recurrent subject groups. These results demonstrate that recurrent subjects after at least 1 month without any antipsychotic drugs show similar plasma acyl-carnitine profiles to first-episode subjects both at baseline and after 8-week follow-up (Table S4).

**Fig. 3:**
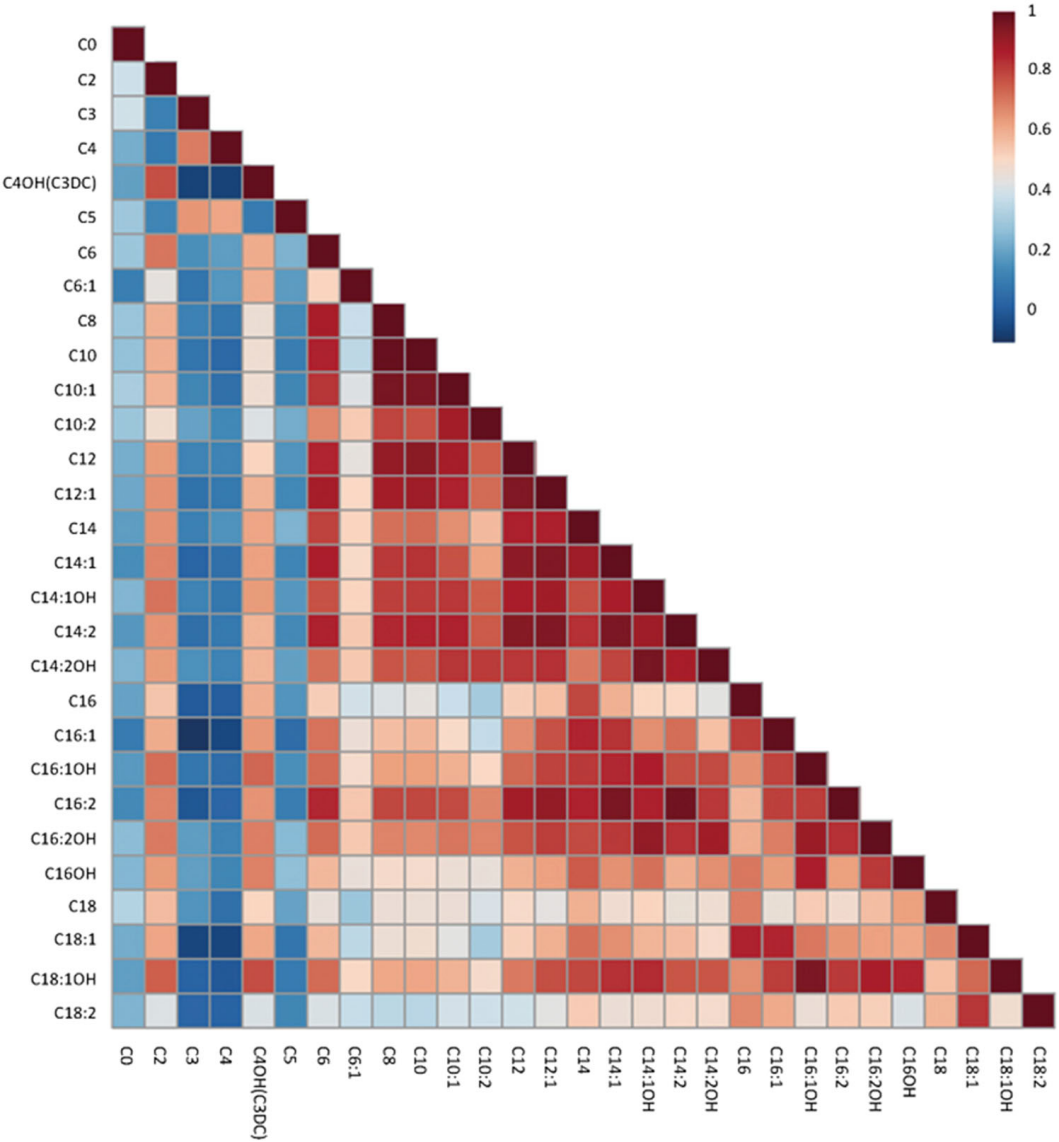
Correlations between the acyl-carnitine levels. Red and blue represent positive and negative correlations, respectively

### Acyl-carnitine panel with best classification performance

The ROC curves were carried out to explore the diagnostic effectiveness of all significant acyl-carnitines between specific groups. In this study, two groups of acyl-carnitines were screened by backward stepwise logistic regression models, which were based on Akaike’s information criterion (AIC). For differentiating subjects with schizophrenia from controls at baseline, a combination of seven acyl-carnitines, including C4OH (C3DC), C8, C10:1, C12, C14:1OH, C14:2, and C16:1, provided the best classification performance with the AUC = 0.926 (95% CI: 0.901–0.952). For differentiating pretreatment from posttreatment subjects, a set of ten acyl-carnitines, including C2, C4, C4OH (C3DC), C10:1, C14:1, C14:1OH, C14:2, C16:1OH, C16:2, and C18:1, was used to build the ROC curve with the AUC = 0.851 (95% CI: 0.810–0.892), which indicated a high accuracy in predicting different groups. Nine acyl-carnitines provided the best classification performance between posttreatment patients and controls (i.e., C2, C8, C10:1, C12, C12:1, C14:1OH, C14:2OH, C16:2OH, and C18) with AUC = 0.950 (95% CI: 0.928–0.972) (Fig. S1).

### Correlations of acyl-carnitines and clinical outcomes

The correlation analysis of acyl-carnitine concentrations was calculated using Spearman correlation analysis, considering subjects both before and after clinical treatment and healthy controls. As shown in Fig. 3, most acyl-carnitines were correlated with each other, especially medium- and long-chain acyl-carnitines. The correlation coefficients among medium- and long-chain acyl-carnitines were strong, with almost all demonstrating R > 0.6 (data available under request). We also performed the correlation analysis of each group. The correlation coefficients of acyl-carnitines were similar in subjects at baseline, after follow-up, and in healthy controls (Figure 3).

Spearman correlation analysis was further applied to examine associations between the baseline levels of the acyl-carnitines and follow-up changes in the clinical variables (Fig. S3). BMI and waist circumference changes were negatively related to several short-chain and medium-chain acyl-carnitines at baseline (*P* < 0.05). Levels of C3 and C5 at baseline were inversely associated with 8-week follow-up changes in FBG among subjects. However, no significant difference was found between acyl-carnitine levels at baseline with regards to the changes of PANSS total scores.

## Discussion

By using metabolomic techniques, our study is the first to accurately quantify acyl-carnitines in relation to schizophrenia within a Chinese population. Our results provide a basis for future studies to determine the role of these mediators in underlying molecular processes and possibly to inform the diagnosis of schizophrenia. When acyl-carnitine profiles were compared between schizophrenia subjects and healthy controls, significantly higher levels of C4-OH(C3-DC) and C16:1 were found, while lower concentrations of C3, C8, C10, C10:1, C10:2, C12, C14:1-OH, C14:2, and C14:2-OH were presented in the subject group after controlling for age, sex, BMI, smoking, and drinking. After the 8-week clinical treatment with mixed antipsychotic medications, all detected acyl-carnitines were significantly different between the two groups. After selection by OPLS-DA VI *P* value>1.0, elevations in a set of short-chain acyl-carnitines (C3 and C4) were found posttreatment. Whether the observed differences are related to the pathoetiology or possible treatment effects of schizophrenia is unknown from our current study. From the observed classification performance of our ROC curve analysis, a set of acyl-carnitines demonstrated an ability to aid differentiating schizophrenia subjects at baseline and after 8-week follow-up for both pretreatment and posttreatment subjects, as well as controls, indicating that bioenergetic abnormalities exist in subjects with schizophrenia. The acyl-carnitine panel demonstrated better diagnostic performance than other diagnostic methods in psychiatry (AUC = 0.85–0.95). Functional near-infrared spectroscopy (fNIRS) is a non-invasive neuroimaging technology that maps the functions of the cerebral cortex by measuring hemodynamics and demonstrates cost-effectiveness^34^. For differentiating schizophrenia and bipolar disorder from major depressive disorder by fNIRS, the AUC is 0.74^35^. For differentiating major depressive disorder from healthy controls by urine monoamine panel, the AUC is 0.66^36^. Consequently, exploring the disrupted acyl-carnitine metabolism from our current results may play a critical role in helping us to deepen our understanding on related bioenergetic pathways of schizophrenia.

Acyl-carnitines are amphiphilic molecules and facilitate mobility throughout cells, which play an essential role in the transport of activated long-chain fatty acids from the cytosol across the mitochondrial membrane toward the mitochondrial matrix, where β-oxidation takes place^37,38^. Similar to small and water-soluble molecules, free carnitines and short-chain acyl-carnitines are easily transported across the plasma membrane and can be used to deliver acyl-groups to various locations with wide ranging functions^11,39^. However, medium-chain and long-chain acyl-carnitines require transporters to cross the plasma membrane, which may result in regulated biological functions^11,40^. In our current study, strong positive correlations among most medium-chain and long-chain acyl-carnitines were found with each other (from C6 to C18, Fig. S3). Our findings suggest that changes in individual acyl-carnitines possibly correlate with each other, especially medium-chain and long-chain acyl-carnitines. Furthermore, acyl-carnitines have important roles in the regulation of cellular energetics and mitochondrial functioning, including roles in the central nervous system (CNS)^41,42^. Previous systematic reviews reported that neuropsychiatric disorders, such as Alzheimer’s disease and autism, often share similar pathologies in mitochondrial dysfunction^43^ regarding energetic metabolic abnormalities.

The comparison between individuals with schizophrenia and healthy controls indicates that nearly all the medium-chain and long-chain acyl-carnitines were lower in the subject group, which may be caused by the activation or inhibition of different transporters in the plasma membrane. Historically, the function of carnitines and their role in the pathophysiology of schizophrenia are not completely clarified. Certain studies have pointed out that acyl-carnitines which have high rates of β-oxidation are found to increase antioxidant activity, regulate immune functions^44^, stimulate the activity of certain proteins and enzymes, and enhance cholinergic neurotransmission^40^. On the other hand, other studies suggest that acyl-carnitines are harmful fatty acid intermediates, which have a role in inducing inhibition of oxidative phosphorylation and increasing apoptosis^45^. Past research also reports that carnitines can be altered under different metabolic conditions^26^; this was illustrated in a population-based study with diabetes that demonstrated carnitines potentially are associated with the pathogenesis of insulin resistance caused by mitochondrial stress, including incomplete fatty acid oxidation and mitochondrial lipid overload^18^. Furthermore, previous studies indicate that treatment with long-chain acyl-carnitines induced insulin resistance and oxidative stress in human skeletal muscles^46^. The conflicting evidence pertaining to acyl-carnitines further sheds light on the fact that more research is needed on this important topic.

After the 8-week follow-up point, a panel of medium-chain and long-chain acyl-carnitines were lower in posttreatment subjects than they were at baseline. Perhaps the observed downregulation of these carnitines after treatment administration is indicative of altered metabolic pathways in energy metabolism, which has been observed in conditions with increased catabolism (e.g., senile dementia, metabolic nerve diseases, tuberculosis, myopathies and cardiomyopathies)^47^. This decrease is also potentially a result of several antipsychotic drugs or mood stabilizers (e.g., valproic acid) that are involved in secondary carnitine deficiency which have been found to reduce levels of acyl-carnitines^48,49^.

Attention needs to be drawn to the increase of C3 and C4 after 8-week treatment (two short-chain acyl-carnitines) due to the possibility of their involvement in different metabolic pathways compared to the other carnitines. Fatty acids can across the plasma membrane with the fatty acid transport proteins. After transportation into the cell by fatty acid transporters, fatty acids are activated by esterification to Acyl-Coenzyme A (Acyl-CoA). Subsequently, Carnitine palmitoyltransferase 1 (CPT1) catalyzes transfer acyl groups from Acyl-CoA to carnitine to produce medium-chain and long-chain acyl-carnitines and free coenzyme A (CoA) under normal conditions^50^. The resulting acyl-carnitines are transported across the inner mitochondrial membrane into the mitochondrion matrix^51,52^. Furthermore, carnitine acetyltransferase (CAT) catalyzes the synthesis of short-chain acyl-carnitines from Acyl-CoA, which is located on the inner mitochondrial membrane, microsomes, and peroxisomes^53,54^. Elevations in a set of short-chain acyl-carnitines (C3 and C4) in posttreatment subjects speculates the dysfunction of CPT1 and CAT; or the deficiency of related acyl-CoA (such as propionyl-CoA carboxylase, isobutyryl-CoA dehydrogenase, isovaleryl-CoA dehydrogenase, and succinyl-CoA synthetase) which also have proven to cause the downregulation of free carnitines^11^.

Acyl-carnitines have previously shown to be reliable biomarkers in the diagnosis or prediction of disease^18,55^ such as type 2 diabetes and atypical myopathy. Moreover, other lines of research indicate that acyl-carnitine supplementation can be considered as a potential therapeutic strategy across several neuropsychiatric disorders (i.e., schizophrenia, autism, depression, etc.) with unique neuroprotective effects including anti-inflammatory and antioxidant benefits^27,43^. Our research results and past published evidence uniformly indicate that acyl-carnitine profiles may be a useful indicator of metabolic changes in schizophrenia, additionally acyl-carnitine supplementation therapies may have accompanying positive potential in the treatment of schizophrenia.

Strengths of our study include the incorporation of high-dimensional datasets with validated profiling tests in a schizophrenia population such that acyl-carnitines with abnormalities in cellular bioenergetics are characterized in schizophrenia. However, the study has several limitations. First, only 69.3% of subjects with schizophrenia were successfully followed. The clinical characteristics and acetyl-carnitines of the followed subjects may differ with those lost to follow-up. Secondly, an 8-week follow-up duration may not be sufficient to observe long-term metabolic and clinical changes (e.g., the development of other medical co-morbidities). Furthermore, given the limited sample size and the heterogeneity of the antipsychotic drugs administered, results should be interpreted with caution. There is also the possibility that changes in plasma acyl-carnitine levels can result from pharmacological intervention. In such cases, the role of plasma acyl-carnitine levels in the etiology of schizophrenia pathogenesis may be limited. Further research on specific antipsychotics are needed to explore pharmacological effects on acyl-carnitines in the future. Finally, the presentation of schizophrenia can be heterogeneous, consequently, our results may not fully capture metabolomic profiles of all presentations of schizophrenia. The reported results in our study provide rationale for the exploration of acyl-carnitines in schizophrenia beyond the Chinese population, as well as larger sample cohorts with longer longitudinal durations.

## Conclusion

In conclusion, our study suggests that acyl-carnitines provide a potential pathway in the pathoetiology of schizophrenia. Acyl-carnitines present novel treatment and screening opportunities for patients with schizophrenia. The current results indicating that bioenergetic abnormalities exist in subjects with schizophrenia. The reported results contained herein warrant further exploration of acyl-carnitine profiles in different populations and large sample cohorts with longer longitudinal durations.

## Supplementary information

Supplemental Materials
