# Supplementary material for: Characterizing acyl-carnitine biosignatures for schizophrenia: a longitudinal pre- and post-treatment study
Source: Transl Psychiatry. 2019 Jan 17;9:19. doi: 10.1038/s41398-018-0353-x (PMC6336814; doi:10.1038/s41398-018-0353-x)
Supplement: Supplementary file 1 — Supplemental Materials [file 41398_2018_353_MOESM1_ESM.docx]

**Supplementary Materials**

**Characterizing Acyl-carnitines Biosignatures for Schizophrenia: A Longitudinal Pre- and Post-Treatment Study**

Bing Cao^1^, Dongfang Wang^1^, Zihang Pan^2^, Elisa Brietzke^2,3^, Roger S. McIntyre^2,4^, Lailai Yan^1,5,6^, Mehala Subramanieapillai^2^, Rodrigo B. Mansur^2^, Natalie Musial^2^, Jing Zeng^1,5,6^, Ninghua Huang^1,5,6^ and Jingyu Wang^1,5,6, *^

^1^ Department of Laboratorial Science and Technology, School of Public Health, Peking University, Beijing 100191, P. R. China

^2^Mood Disorders Psychopharmacology Unit, Toronto Western Hospital, University Health Network, Toronto, ON, Canada.

^3^ Department of Psychiatry, Federal University of São Paulo, São Paulo, SP, Brazil.

^4^ Brain and Cognition Discovery Foundation, Toronto, ON, Canada

^5^ Beijing Key Laboratory of Toxicological Research and Risk Assessment for Food Safety, Beijing 100191, P.R. China

^6^ Peking University Medical and Health Analysis Center, Peking University, Beijing 100191, P.R. China

***Corresponding Author:**

Jing-Yu Wang, PhD, Department of Laboratorial Science and Technology, School of Public Health, Peking University, 38 Xue-Yuan Road, Haidian District, Beijing 100191, P.R. China. Tel/Fax: + 86-10-82801107, E-mail: [wjy@bjmu.edu.cn](mailto:wjy@bjmu.edu.cn)

**Contents**

**Table S1.** Clinical treatment therapies of Study Participants

| **Categories** | **Drugs** | **No. of Subjects (n, %)** |
| --- | --- | --- |
| First generation antipsychotics | Haloperidol | 7(4.49) |
|  | Chlorpromazine | 1(0.64) |
|  | Promethazine | 6(3.85) |
| Second generation antipsychotics | Olanzapine | 31(19.87) |
|  | Risperidone | 53(33.97) |
|  | Clozapine | 40(25.64) |
|  | Quetiapine | 66(42.31) |
|  | Ziprasidone | 31(19.87) |
|  | Aripiprazole | 28(17.95) |
|  | Perpentine | 6(3.85) |
|  | Sulpiride | 2(1.28) |
| Anxiolytics | Lorazepam | 46(29.49) |
|  | Clonazepam | 7(4.49) |
|  | Alprazolam | 12(7.69) |
| Anticonvulsants | Magnesium valproate | 21(13.46) |
|  | Sodium valproate | 24(15.38) |
| Anti-Tremor Medications | Phenanthrene | 33(21.15) |

**Table S2.** Qualitative and quantitative of 29 acyl-carnitines

| **Classification** | **Acyl-carnitines** | **Biochemical Name** | **Quality Type** | | **Quantitated by** |
| --- | --- | --- | --- | --- | --- |
|  |  |  | **Valid** | **Semi** |  |
| Free carnitine | C0 | Carnitine | √ |  | ^2^H_9_-Carnitine (C0) |
| Short-chain acyl-carnitines | C2 | Acetylcarnitine | √ |  | ^2^H_3_-Acetylcarnitine (C2) |
|  | C3 | Propionyl-carnitine | √ |  | ^2^H_3_-Propionylcarnitine (C3) |
|  | C4 | Butyryl-carnitine | √ |  | ^2^H_3_-Butyrylcarnitine (C4) |
|  | C4-OH(C3-DC) | Malonyl-carnitine  (Hydroxy-butyrylcarnitine) |  | √ | ^2^H_3_-Butyrylcarnitine (C4) |
|  | C5 | Valeryl-carnitine | √ |  | ^2^H_9_-Isovalerylcarnitine(C5 ) |
| Medium-chain acyl-carnitines | C6 | Hexanoylcarnitine |  | √ | ^2^H_9_-Isovalerylcarnitine(C6 ) |
|  | C6:1 | Hexenoyl-carnitine |  | √ | ^2^H_9_-Isovalerylcarnitine(C6 ) |
|  | C8 | Octanoylcarnitine | √ |  | ^2^H_3_-Octanoylcarnitine(C8 ) |
|  | C10 | Decanoyl-carnitine |  | √ | ^2^H_3_-Octanoylcarnitine(C8 ) |
|  | C10:1 | Decenoyl-carnitine |  | √ | ^2^H_3_-Octanoylcarnitine(C8) |
|  | C10:2 | Decadienyl-carnitine |  | √ | ^2^H_3_-Octanoylcarnitine(C8) |
| Long-chain acyl-carnitines | C12 | Dodecanoyl-carnitine |  | √ | ^2^H_3_-Octanoylcarnitine(C8) |
|  | C12:1 | Dodecenoyl-carnitine |  | √ | ^2^H_3_-Octanoylcarnitine(C8) |
|  | C14 | Tetradecanoyl-carnitine | √ |  | ^2^H_9_-Myristoylcarnitine(C14 ) |
|  | C14:1 | Tetradecenoyl-carnitine |  | √ | ^2^H_9_-Myristoylcarnitine(C14 ) |
|  | C14:1-OH | Hydroxytetra-decenoyl-carnitine |  | √ | ^2^H_9_-Myristoylcarnitine(C14 ) |
|  | C14:2 | Tetradecadienyl-carnitine |  | √ | ^2^H_9_-Myristoylcarnitine(C14 ) |
|  | C14:2-OH | Hydroxytetra-decadienyl-carnitine |  | √ | ^2^H_9_-Myristoylcarnitine(C14 ) |
|  | C16 | Hexadecanoyl-carnitine | √ |  | ^2^H_3_-Palmitoylcarnitine (C16 ) |
|  | C16:1 | Hexa-decenoyl-carnitine |  | √ | ^2^H_3_-Palmitoylcarnitine (C16 ) |
|  | C16:1-OH | Hydroxyhexa-decenoyl-carnitine |  | √ | ^2^H_3_-Palmitoylcarnitine (C16 ) |
|  | C16:2 | Hexadeca-dienylcarnitine |  | √ | ^2^H_3_-Palmitoylcarnitine (C16 ) |
|  | C16:2-OH | Hydroxyhexa-decadienyl-carnitine |  | √ | ^2^H_3_-Palmitoylcarnitine (C16 ) |
|  | C16-OH | Hydroxyhexa-decanoyl-carnitine |  | √ | ^2^H_3_-Palmitoylcarnitine (C16 ) |
|  | C18 | Octadecanoyl-carnitine |  | √ | ^2^H_3_-Palmitoylcarnitine (C16 ) |
|  | C18:1 | Octadecenoyl-carnitine |  | √ | ^2^H_3_-Palmitoylcarnitine (C16 ) |
|  | C18:1-OH | Hydroxyocta-decenoyl-carnitine |  | √ | ^2^H_3_-Palmitoylcarnitine (C16 ) |
|  | C18:2 | Octadecadienyl-carnitine |  | √ | ^2^H_3_-Palmitoylcarnitine (C16 ) |

**Table S3.** Acyl-carnitines levels of patients of schizophrenia at baseline and healthy control, pretreatment and posttreatment patients

| **Acylcarnitines (µmol/L)**;  median (IQR) | **Schizophrenia** | **Control** |  | **Pretreatment** | **Posttreatment** |
| --- | --- | --- | --- | --- | --- |
|  | **n=225** | **n=175** |  | **(N=156)** | **(N=156)** |
| C0 | 45.58(33.11-58.97) | 53.17(38.91-65.4) |  | 45.74(34.22-60.09) | 38.51(28.57-56.16) |
| C2 | 10.36(7.01-15.14) | 11.16(9.26-13.24) |  | 10.29(6.92-15.18) | 7.62(5.98-9.78) |
| C3 | 0.42(0.31-0.59) | 0.55(0.45-0.72) |  | 0.42(0.31-0.58) | 0.55(0.40-0.77) |
| C4 | 0.17(0.13-0.23) | 0.21(0.17-0.27) |  | 0.17(0.13-0.23) | 0.25(0.18-0.40) |
| C4-OH(C3-DC) (10^-2^) | 3.57(1.9-7.85) | 2.56(1.79-3.68) |  | 3.62(1.95-7.81) | 1.83(1.34-2.79) |
| C5 (10^-2^) | 8.48(6.38-11.37) | 8.97(6.96-11.82) |  | 8.3(6.16-11.65) | 9.94(7.33-12.99) |
| C6 (10^-2^) | 7.14(4.58-11.1) | 7.76(6.04-11.04) |  | 7.11(4.02-10.68) | 5.49(3.57-8) |
| C6:1 (10^-2^) | 0.69(0.5-0.97) | 0.62(0.44-0.81) |  | 0.67(0.48-1.02) | 0.58(0.43-0.85) |
| C8 (10^-2^) | 13.68(7.25-24.2) | 23.1(15.98-34.69) |  | 13.78(7.13-24.22) | 9.11(6.19-16.04) |
| C10 (10^-2^) | 17.2(7.86-29.64) | 27.84(18.44-41.46) |  | 17.9(7.83-28.41) | 9.61(6.58-18.74) |
| C10:1 (10^-2^) | 18.9(10.54-30.6) | 33.97(25.19-46.99) |  | 19.78(10.51-31.22) | 13.07(8.35-21.66) |
| C10:2 (10^-2^) | 1.95(1.34-2.77) | 3.3(2.31-4.46) |  | 1.97(1.24-2.9) | 1.71(1.06-2.66) |
| C12 (10^-2^) | 4.49(2.42-7.55) | 6.48(4.57-9.3) |  | 4.7(2.31-7.5) | 3.35(2.05-5.62) |
| C12:1 (10^-2^) | 7.99(3.88-11.92) | 8.74(6.23-12) |  | 8.38(3.97-11.83) | 4.67(2.75-8.13) |
| C14 (10^-2^) | 1.34(0.97-2) | 1.34(1.1-1.78) |  | 1.36(0.93-2.01) | 1.17(0.88-1.61) |
| C14:1 (10^-2^) | 6.76(3.62-10.7) | 7.23(5.04-9.51) |  | 6.85(3.63-10.23) | 4.01(2.3-7.23) |
| C14:1-OH (10^-2^) | 0.59(0.3-0.86) | 0.74(0.54-1.03) |  | 0.59(0.28-0.83) | 0.36(0.2-0.57) |
| C14:2 (10^-2^) | 7.58(3.75-11.53) | 8.98(6.42-12.8) |  | 7.62(3.68-11.4) | 4.5(2.48-8.41) |
| C14:2-OH (10^-2^) | 0.28(0.15-0.45) | 0.39(0.29-0.58) |  | 0.28(0.14-0.45) | 0.19(0.09-0.34) |
| C16 (10^-2^) | 8.7(7.19-11.31) | 7.95(6.59-9.48) |  | 9.32(7.32-11.47) | 7.64(6.2-9.52) |
| C16:1 (10^-2^) | 2.81(1.96-3.82) | 2.19(1.65-2.77) |  | 2.92(1.97-3.98) | 1.99(1.34-2.77) |
| C16:1-OH (10^-2^) | 0.19(0.12-0.3) | 0.19(0.13-0.25) |  | 0.19(0.11-0.3) | 0.13(0.07-0.21) |
| C16:2 (10^-2^) | 1.36(0.8-1.98) | 1.36(1.04-1.84) |  | 1.37(0.83-1.9) | 0.81(0.48-1.4) |
| C16:2-OH (10^-2^) | 0.47(0.3-0.66) | 0.53(0.4-0.68) |  | 0.48(0.3-0.65) | 0.34(0.22-0.53) |
| C16-OH (10^-2^) | 0.15(0.09-0.23) | 0.15(0.11-0.21) |  | 0.15(0.09-0.23) | 0.13(0.08-0.18) |
| C18 (10^-2^) | 1.97(1.6-2.58) | 2.22(1.84-2.66) |  | 1.96(1.62-2.6) | 1.69(1.28-2.11) |
| C18:1 (10^-2^) | 11.62(9.35-14.97) | 10.4(8.76-12.86) |  | 12.23(9.84-15.07) | 8.2(5.62-11.2) |
| C18:1-OH (10^-2^) | 0.21(0.12-0.3) | 0.19(0.14-0.24) |  | 0.21(0.12-0.3) | 0.12(0.06-0.19) |
| C18:2 (10^-2^) | 12.78(10.73-16.4) | 13.1(10.65-15.9) |  | 13.31(11.01-16.46) | 9.91(7.95-14.44) |

**Supplemental Table 4.** Basic Characteristics and acyl-carnitines of first-episode and recurrent subjects of schizophrenia

| **Variables** | **Pretreatment** | | ***P* value**^‡^ | **Posttreatment** | | ***P* value**^#^ |
| --- | --- | --- | --- | --- | --- | --- |
|  | **First-episode** | **Recurrent** |  | **First-episode** | **Recurrent** |  |
|  | ***n=*40** | ***n*=185** |  | **n=34** | **n=122** |  |
| **Age**(years); mean (SD) | 32.51(9.7)* | 38.12(10.83) | **0.003** | 30.97(11.05) | 36.88(10.88) | **0.006** |
| **Gender** (male/female) | 17/23 | 73/110 | **0.040** | 13/21 | 49/73 | **0.041** |
| **PANSS scores**; mean (SD) |  |  |  |  |  |  |
| PANSS total | 87.04(17.93) | 89.96(17.12) | 0.439 | 47.85(8.56) | 50.46(12.27) | 0.307 |
| PANSS positive | 20.5(7.88) | 22.08(8.1) | 0.369 | 9.85(3.68) | 9.98(4.08) | 0.877 |
| PANSS negative | 19.12(8.43) | 21.42(7.97) | 0.191 | 13.76(4.79) | 13.63(5.36) | 0.908 |
| General psychopathology | 42.73(13.11) | 42.51(12.8) | 0.938 | 26.28(6.81) | 25.54(8.19) | 0.673 |
| **BMI** (kg/m2); mean (SD) | 23.24(3.13) | 24.02(4.12) | 0.328 | 24.16(3.24) | 24.85(3.77) | 0.358 |
| **Waist** (cm); mean (SD) | 85.23(10.03) | 89.33(12.62) | 0.096 | 87.53(9.31) | 91.29(11.58) | 0.102 |
| **FBG** (mmol/L); mean (SD) | 5.28(0.74) | 5.84(1.96) | 0.075 | 5.08(0.65) | 5.25(1.07) | 0.401 |
| **TG** (mmol/L); mean (SD) | 1.07(0.64) | 1.3(0.94) | 0.127 | 1.74(0.9) | 2.03(1.21) | 0.229 |
| **TC**(mmol/L); mean (SD) | 4.32(0.89)* | 4.73(1.13) | **0.032** | 4.3(0.85) | 4.72(0.92) | **0.032** |
| **HDL** (mmol/L); mean (SD) | 1.43(0.27) | 1.43(0.31) | 0.965 | 1.32(0.3) | 1.36(0.32) | 0.592 |
| **LDL** (mmol/L); mean (SD) | 2.11(0.39)* | 2.28(0.46) | **0.029** | 2.13(0.53) | 2.33(0.43) | **0.034** |
| **VLDL** (mmol/L); mean (SD) | 0.49(0.3) | 0.6(0.03) | 0.130 | 0.8(0.41) | 0.93(0.55) | 0.223 |
| **Acyl-carnitines (µmol/L)**; median (IQR) |  |  |  |  |  |  |
| C0 | 42.26(32.15-56.23) | 45.59(33.29-61.05) | 0.623 | 34.75(26.17-47.16) | 41.04(30.54-57.04) | 0.083 |
| C2 | 11.18(6.44-18.39) | 10.25(7.16-14.74) | 0.763 | 7.08(5-9.45) | 7.83(6.06-9.98) | 0.358 |
| C3 | 0.32(.24-0.45) | 0.44(0.33-0.61) | 0.005 | 0.48(0.34-0.67) | 0.59(0.42-0.80) | 0.009 |
| C4 | 0.15(0.12-0.16) | 0.18(0.13-0.24) | 0.009 | 0.20(0.15-0.26) | 0.27(0.19-0.45) | 0.017 |
| C4-OH(C3-DC) (10^-2^) | 5.13(1.54-10.81) | 3.5(1.93-7.65) | 0.490 | 1.6(1.19-2.29) | 1.91(1.36-2.81) | 0.306 |
| C5 (10^-2^) | 8.11(5.56-10.02) | 8.73(6.53-11.81) | 0.297 | 8.32(6.09-11.39) | 10.03(7.39-13.2) | 0.029 |
| C6 (10^-2^) | 6.43(3.84-9.66) | 7.49(4.93-11.49) | 0.167 | 5.21(3.09-6.64) | 5.64(3.71-8.29) | 0.358 |
| C6:1 (10^-2^) | 0.61(0.47-0.87) | 0.71(0.51-0.99) | 0.360 | 0.53(0.36-0.76) | 0.6(0.45-0.89) | 0.078 |
| C8 (10^-2^) | 11.96(6.09-19.25) | 14(7.35-24.32) | 0.173 | 8.98(5.62-16.69) | 9.11(6.39-15.86) | 0.866 |
| C10 (10^-2^) | 12.99(5.92-24.51) | 18.02(8.19-30.85) | 0.147 | 9.71(5.66-19.01) | 9.61(6.63-18.85) | 0.694 |
| C10:1 (10^-2^) | 17.63(8.8-26.53) | 20.14(10.75-30.74) | 0.156 | 13.04(5.83-20.87) | 13.07(8.62-21.77) | 0.969 |
| C10:2 (10^-2^) | 1.81(1.08-2.38) | 1.97(1.37-2.9) | 0.126 | 1.56(1.03-3.04) | 1.81(1.05-2.64) | 0.916 |
| C12 (10^-2^) | 3.64(1.62-7.81) | 4.65(2.53-7.46) | 0.138 | 2.99(1.85-5.08) | 3.39(2.09-5.67) | 0.969 |
| C12:1 (10^-2^) | 6.75(3.24-11.29) | 8.22(4.15-12.11) | 0.126 | 4.14(2.37-8.33) | 5.15(2.81-8.07) | 0.511 |
| C14 (10^-2^) | 1.13(0.75-1.59) | 1.43(1.01-2.02) | 0.118 | 0.97(0.69-1.57) | 1.21(0.93-1.62) | 0.198 |
| C14:1 (10^-2^) | 6.26(2.62-9.88) | 6.85(3.78-10.98) | 0.211 | 4.02(1.93-6.67) | 3.99(2.31-7.47) | 0.781 |
| C14:1-OH (10^-2^) | 0.61(0.25-0.85) | 0.58(0.31-0.84) | 0.433 | 0.32(0.18-0.5) | 0.37(0.21-0.59) | 0.973 |
| C14:2 (10^-2^) | 6.82(3.03-11.45) | 7.76(4.05-11.29) | 0.227 | 4.41(2.35-7.55) | 4.5(2.51-8.58) | 0.842 |
| C14:2-OH (10^-2^) | 0.30(0.12-0.48) | 0.27(0.15-0.45) | 0.311 | 0.14(0.08-0.32) | 0.2(0.09-0.34) | 0.903 |
| C16 (10^-2^) | 7.6(6.35-10.21) | 9.11(7.41-11.7) | 0.046 | 6.79(5.74-8.26) | 7.78(6.5-9.56) | 0.092 |
| C16:1 (10^-2^) | 2.3(1.82-3.44) | 2.92(2-4.09) | 0.223 | 1.81(1.14-2.73) | 2.01(1.35-2.78) | 0.383 |
| C16:1-OH (10^-2^) | 0.16(0.09-0.33) | 0.19(0.12-0.29) | 0.397 | 0.09(0.06-0.17) | 0.13(0.08-0.23) | 0.315 |
| C16:2 (10^-2^) | 1.23(0.58-2.1) | 1.39(0.83-1.93) | 0.308 | 0.75(0.51-1.28) | 0.82(0.47-1.42) | 0.856 |
| C16:2-OH (10^-2^) | 0.46(0.23-0.71) | 0.48(0.31-0.65) | 0.539 | 0.3(0.19-0.4) | 0.36(0.22-0.54) | 0.582 |
| C16-OH (10^-2^) | 1.41(0.62-2.37) | 1.55(1.02-2.27) | 0.435 | 0.88(0.65-1.54) | 1.37(0.81-2) | 0.128 |
| C18 (10^-2^) | 1.85(1.4-2.57) | 1.98(1.62-2.59) | 0.426 | 1.47(1.1-2.03) | 1.76(1.33-2.14) | 0.151 |
| C18:1 (10^-2^) | 10.57(9.33-13.28) | 11.93(9.23-15.38) | 0.140 | 7.39(5.23-10.48) | 8.54(5.84-11.78) | 0.516 |
| C18:1-OH (10^-2^) | 0.19(0.11-0.33) | 0.21(0.12-0.29) | 0.473 | 0.09(0.06-0.17) | 0.13(0.07-0.21) | 0.534 |
| C18:2 (10^-2^) | 11.61(10.8-15.36) | 12.83(10.67-16.85) | 0.298 | 9.91(7.08-13.09) | 9.91(8.03-14.94) | 0.645 |

^#^*P*-values were calculated by adjusting age, gender, BMI. PANSS, the Positive and Negative Syndrome Scale; BMI, body mass index; FBG, fasting blood glucose; TG, triglyceride; TC, total cholesterol; HDL, high density lipoprotein; LDL, low density lipoprotein; VLDL, very low density lipoprotein; SD, standard deviation; IQR, interquartile range.

**Figure Legends**

**Supplemental Figure 1.** ROC curves illustrate the classification performance of biomarker panels for distinguishing between (A) schizophrenia subjects and healthy controls, AUC=0.952 (95%CI: 0.932-0.972); (B) posttreatment and pretreatment subjects, AUC=0.871(95%CI: 0.837-0.905); (C) pretreatment subjects and healthy controls, AUC=0.950 (95%CI: 0.928-0.972).

**Supplemental Figure 2.** Associations between the baseline levels of the acyl-carnitines (columns) and follow-up changes (rows) in the clinical variables. Red and blue represent positive and negative correlations, respectively. + Correlation is positive significant, - Correlation is negative significant (p<0.05). BMI, Body Mass Index; FBG, Fasting Blood Glucose; TG, Triglyceride; TC, Total Cholesterol; HDL, High Density Lipoprotein; LDL, Low Density Lipoprotein; VLDL, Very Low Density Lipoprotein; P score, Positive Scores of PANSS Scale; N score, Negative Scores of PANSS Scale; T score, Total Scores of PANSS.


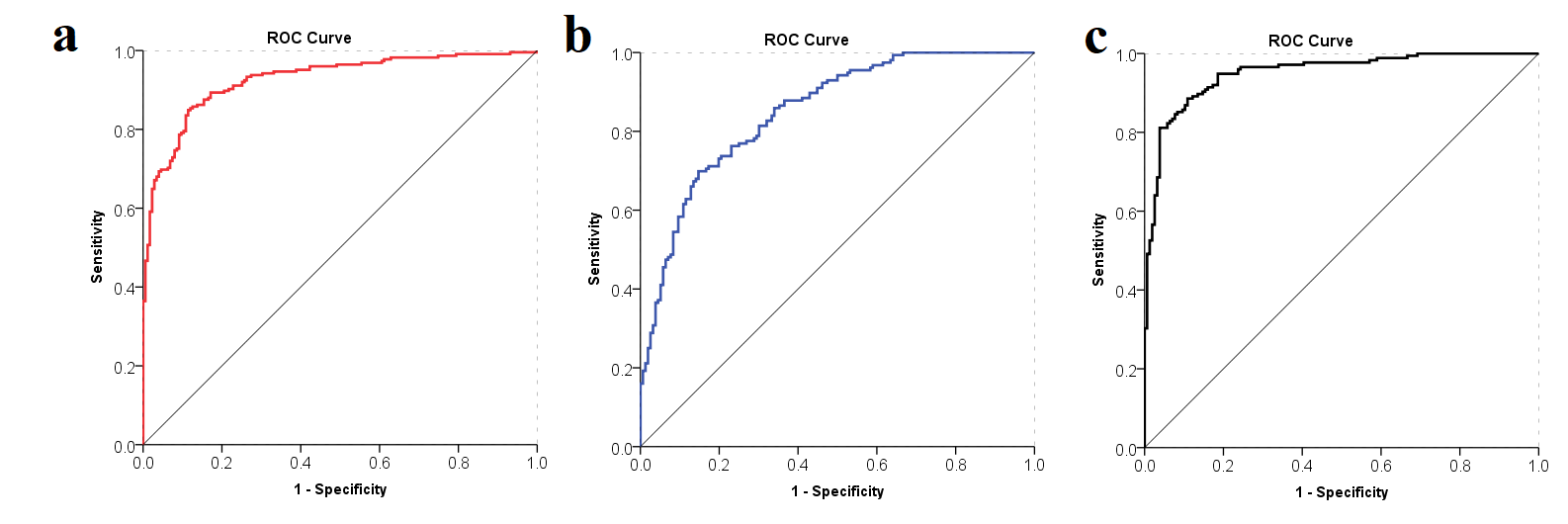


**Supplemental Figure 1.**

**
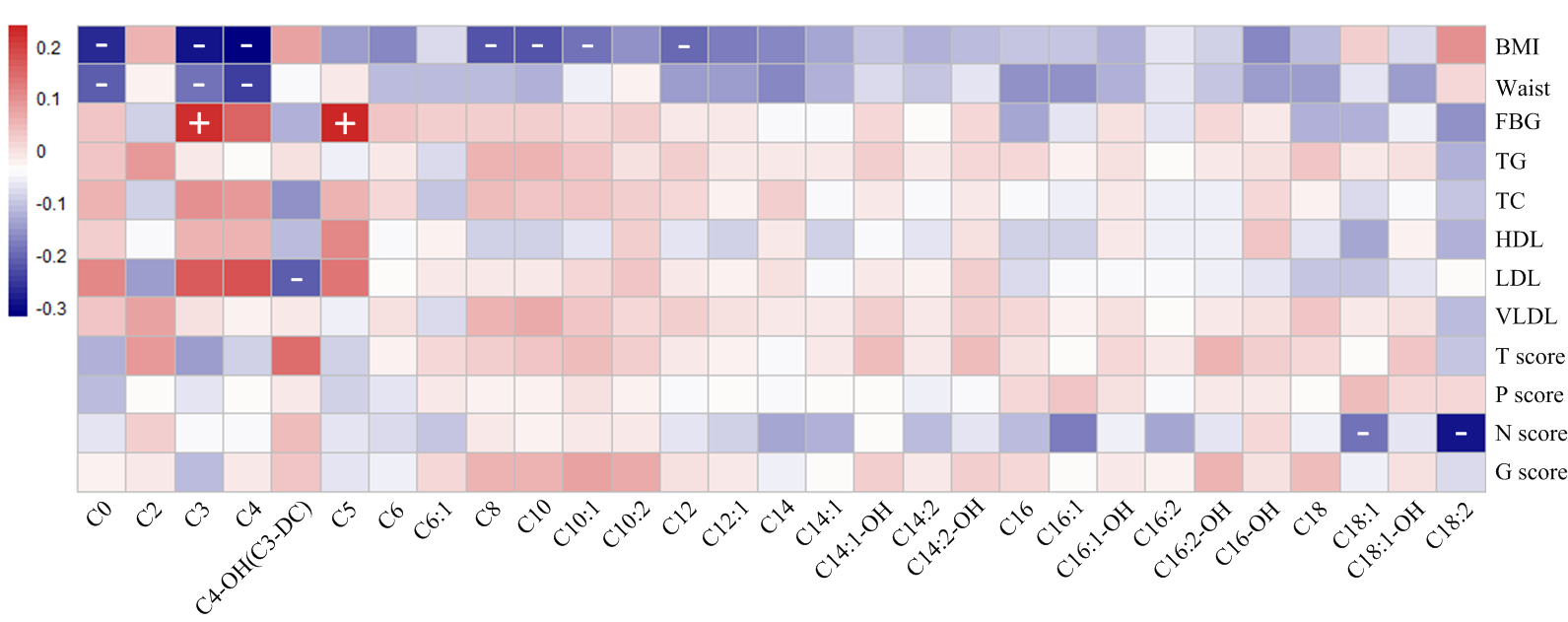
**

**Supplemental Figure 2.**
